# A tool for mass-screening of paragonimiasis: an enzyme-linked immunosorbent assay with urine samples

**DOI:** 10.1186/s41182-016-0019-4

**Published:** 2016-06-29

**Authors:** Xu Guang Qiu, Fukumi Nakamura-Uchiyama, Yukifumi Nawa, Makoto Itoh

**Affiliations:** Department of Microbiology and Immunology, Aichi Medical University School of Medicine, Nagakute, Aichi 480-1195 Japan; Department of Pathogen, Infection and Immunity, Nara Medical University, Kashihara, Nara 634-8521 Japan; Research Affairs, Faculty of Medicine, Khon Kaen University, Khon Kaen, 20004 Thailand

**Keywords:** Paragonimiasis, Diagnosis, Mass-screening, Urine, ELISA

## Abstract

**Background:**

Paragonimiasis is one of the foodborn trematodiases and number of the patients was estimated to be about 23 million around the world. To obtain good compliance of people for the surveillance of paragonimiasis, an enzyme-linked immunosorbent assay (ELISA) for the diagnosis of paragonimiasis with unconcentrated urine samples was developed.

**Results:**

*Paragonimus westermani* antigen specific IgG and IgG4 were detected in urine samples from paragonimiasis patients and the levels correlated well with those detected in the paired serum samples. Cross-reactions observed among other trematodiasis and a tuberculosis patient with the antigen specific IgG were much reduced by detecting the antigen specific IgG4; 9.2 % to 2.3 %.

**Conclusions:**

The ELISA with urine samples, which are collected safely and easily, will be a useful tool for a mass-screening of paragonimiasis.

## Background

Paragonimiasis is a parasitic disease caused by several lung fluke species in the genus *Paragonimus*. Infections of the flukes have been mainly reported from Asian countries [1–5], but emerging cases have also been reported from African [6, 7] and American countries [8, 9], where people eat the intermediate hosts, fresh water crabs, and crayfish containing metacercariae or meat of the reservoir hosts containing immature flukes. About 23 million people around the world are estimated to be infected with the lung flukes [10]. Paragonimiasis is often misdiagnosed as pulmonary tuberculosis because the similarities of their clinical manifestations such as chronic productive cough, hemoptysis, and chest pain [11–16]. Praziquantel is a drug of choice with high cure rate. Although diagnosis of patients with the symptoms is important, active surveillance to find out the endemic foci is necessary for control of the disease [17]. As the symptom of paragonimiasis is not always severe, it will be sometimes difficult to obtain good compliance of the people. Mass-screening methods which are accepted by the residents are required.

For the immune-serological screening, blood samples are usually used. However, collection of urine samples is much more easy, safe and non-invasive. Enzyme-linked immunosorbent assay, ELISA, which detect antigen-specific antibodies in urine samples have been developed for schistosomiasis, lymphatic filariasis, visceral leishmaniasis, and echinococcosis [18–21]. For paragonimiasis, parasite-specific antibodies were shown in urine samples of rats infected with *P. ohirai* [22].

In this study, we report an ELISA to detect antibodies specific to *Paragonimus* antigens in urine samples from paragonimiasis patients which will be useful for the survey of the lung fluke infections.

## Methods

### Urine and serum samples

Twenty-seven paired urine and serum samples and eight urine samples obtained from 19 paragonimiasis westermani patients in Japan were used. Most of them were residents of Miyazaki Prefecture in Kyusyu district, Japan. They were confirmed as paragonimiasis from their eating histories and clinical and serological diagnosis: eosinophilia, coughing, abnormal results of image diagnosis with X-ray and/or CT, and *P. westermani-*specific IgG positive. Urine samples from patients with other trematodiasis were also used; 39 schistosomiasis japonicum, 24 opisthorchiasis viverrini, 16 patients infected with intestinal flukes, and 13 samples from tuberculosis patients. Urine samples from 39 healthy Japanese were used as negative controls. All the serum samples were kept at −20 °C. Urine samples collected at study fields were added with sodium azide (finally 0.1 %), transported to the laboratory at ambient temperature and kept at 4 °C until use. The urine samples collected at hospitals were transported to the laboratory at −20 or 4 °C, then added with sodium azide and kept at 4 °C until use.

### Preparation of *P. westermani* adult worm (AW) antigens

Adult worms of *P. westermani* obtained from experimentally infected rats were homogenized in 1/15 M phosphate-buffered saline (PBS), pH 7.4, containing protease inhibitor cocktail (Sigma-Aldrich Japan K.K., Tokyo). The homogenate was centrifuged at 1600 × g for 20 min at 4 °C, and the supernatant was used as AW antigens. They were kept in aliquots at −80 °C until used.

### ELISA for antibody detection in urine and serum samples

A 96-well microtiter plate (MaxiSorp™, Nunc Denmark) was coated with 10 μg/ml of the *Paragonimus westermani* adult worm (AW) antigens at 4 °C overnight. After washing with a washing buffer (Tween-PBS: 0.05 % Tween 20 in 1/15 M PBS, pH 7.4), the plate was blocked with a casein buffer (1 % casein in 0.05 M Tris–HCl buffer with 0.15 M NaCl, pH 7.6) for 2 h at room temperature. Urine samples (four times diluted by PBS, 100 μl) or serum samples (200,000 times diluted by PBS, 100 μl) were applied to the wells, and the plate was incubated at 37 °C for 2 h for IgG, while at 25 °C overnight for IgG4 detection. After washing the plate four times with the washing buffer, 100 μl of anti-human IgG (4000 times diluted) or anti-human IgG4 (1000 times diluted) conjugated with horseradish peroxidase (Caltag Lab. Inc., San Francisco, CA) was added and incubated at 37 °C for 1 h. ABTS peroxidase substrate (KPL Inc., Gaithersburg, MD) was used for colorization. Optical density was measured at 415 nm, with 492 nm as a reference.

Antibody levels were expressed as units (U) on the basis of a standard curve. To construct the standard curve, threefold serially diluted (1:9000 to 1:6,561,000 for IgG and 1:111 to 1:80,919 for IgG4) pooled sera of paragonimiasis patients were applied to each ELISA plate. As antibody units, a value of 7290 U was arbitrarily assigned to 1:9000 and 1:111 dilutions for IgG and IgG4 detection, respectively. Antibody units >7290 U were regarded as 7290 U. Cutoff point for IgG and IgG4 urine ELISA were obtained from ROC curves constructed; they are 178 and 17 U, respectively.

## Results

### Anti-AW antibodies in urine from paragonimiasis patients

Anti-AW IgG and IgG4 were examined in urine samples from paragonimiasis patients by the ELISA in 27 totally paired urine and serum samples from 19 paragonimiasis patients. Anti-AW antibody levels of urine and serum samples correlated well; *r* = 0.75, (95 % confidential interval [CI] 0.52–0.88) for IgG and *r* = 0.88, (95 % CI, 0.75–0.94) for IgG4.

### Sensitivity and specificity of the urine ELISA

**Fig. 1:**
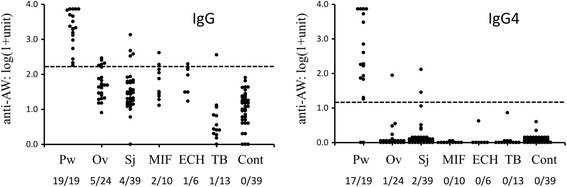
IgG and IgG4 to *P. westermani* adult worm antigens detected in urine samples. Urine samples are from patients infected with, *Pw P. westermani*, *Ov O. viverrini*, *Sj S. japonicum*, *MIF* minute intestinal flukes, *ECH Echinostoma*, and *Mycobacterium tuberculosis*. Control sera were from Japanese healthy controls. *Dotted lines* indicate cutoff. *Numbers* under the abbreviations are the number of positives/examined

All urine samples collected from 19 paragonimiasis patients before treatment were positive with IgG. On the other hand, 17 (90 %) samples were positive with IgG4 ELISA (Fig. 1). Anti-AW IgG and IgG4 levels of 131 urine samples from other trematodiases, tuberculosis patients, and healthy Japanese controls are shown in Fig. 1. Cross-reactions of the IgG ELISA were observed with urine samples from patients infected with trematodes; *O. viverrini* (5/24, 21 %), *S. japonicum* (4/39, 10 %), minute intestinal flukes (2/10, 20 %), and *Echinostoma* spp. (1/6, 17 %). These cross-reactions were much reduced when antigen-specific IgG4 were detected; only 1/24 of opisthorchiasis and 2/39 of schistosomiasis patients were positive, and the positive among the tuberculosis cases by IgG ELISA became negative. Japanese healthy controls were negative with both IgG and IgG4 ELISA. Among the 19 urine samples collected before treatment, two were negative with anti-AW IgG4. Two more paired serum and urine samples from one of the patients with negative IgG4 collected 2 and 4 months after the treatment were examined: anti-AW IgG were positive with both serum and urine samples, but IgG4 were all negative. Another anti-AW negative IgG did not have paired serum. The positive predictive values (PPV) of the IgG and IgG4 ELISA tests were 0.59 (95 % CI, 0.51–0.59) and 0.85 (95 % CI, 0.70–0.92), and the negative predictive values (NPV) of the both tests were 1.0 (95 % CI, 0.98–1.0) and 0.98 (95 % CI, 0.96–1.0), respectively.

### Changes of the anti-AW antibody levels after treatment

The paired urine and serum samples collected from 11 patients with varying periods after the treatment, 1 to 12 months, were also examined for their anti-AW antibody levels. Decrease of anti-AW IgG and IgG4 in urine samples was observed in nine patients, although they were still positive. Peak of the anti-AW IgG and IgG4 levels was observed after the treatment in the other two patients. The similar transition of the antibody levels were observed with the paired serum samples.

## Discussion

Although definitive diagnosis of paragonimiasis is to find the parasite eggs in sputum or stool samples, they are not always detected [23]. ELISA to detect the parasite-specific antibodies in serum is a highly sensitive and specific diagnostic method for paragonimiasis [24]. The antibody detection test itself cannot distinguish past and present infections. However, the probability can be increased when it is combined with clinical manifestations. This study showed that anti-AW antibodies can be detected in urine samples from paragonimiasis patients.

The IgG urine ELISA showed cross-reactions with samples from other trematodiases. Wongkhan et al. showed that IgG1, IgG2, and IgG3 subclass IgG cause the cross-reactions and some TB patients produce IgG2 and IgG3 subclass IgG [25] and no cross-reaction was observed when IgG4 was detected. Our study also showed that IgG4 urine ELISA reduced the cross-reaction; the cross-reactions with samples from other trematodiases were reduced from 15 to 3.8 %. Furthermore, the troublesome cross-reaction with a sample from tuberculosis patient was diminished. Application of recombinant antigens or a synthetic peptide [26] will increase the sensitivity and specificity of this urine ELISA. Decrease of the antibody levels in urine samples observed after treatment will be an indicator of the therapeutic effect.

Through our survey with the urine samples [27–29], we have recognized that the easy and safe urine collection was accepted by the people who participated with good compliance, even schoolchildren. This is important when active surveys are conducted to find endemic foci where people are indifferent to control the disease. The urine ELISA developed for paragonimiasis will facilitate the survey.

## Conclusions

In this study, we developed an ELISA to detect antigen-specific IgG4 in urine samples for the diagnosis of paragonimiasis with high sensitivity and specificity. As the urine samples are easily and safely collected, the ELISA is useful for the mass-survey of the food-born lung fluke infections which distribute over countries in Asia, Africa, and Central and South America where people consume the raw or insufficiently heated second intermediate hosts and/or paratenic hosts.
